# Clinical and optic coherence tomography findings of focal choroidal excavation in Chinese patients

**DOI:** 10.1186/1471-2415-14-63

**Published:** 2014-05-06

**Authors:** Jie Guo, Lu Zhong, Chunhui Jiang, Xin Zhou, Gezhi Xu, Wenji Wang, Yuliang Wang

**Affiliations:** 1 Department of Ophthalmology, Eye & ENT Hospital, Fudan University, Fenyang Road No. 83, Shanghai 200031, China; 2 Department of Ophthalmology, Jiangsu Province Chinese Medicine Hospital, Nanjing, Jiangsu, China

**Keywords:** Choroidal excavation, Optic coherence tomography, Central serous chorioretinopathy, Neovascularization

## Abstract

**Background:**

To describe the clinical and optical coherence tomography (OCT) features of focal choroidal excavation in Chinese patients.

**Methods:**

Retrospectively, thirty-seven eyes (in 31 patients) that demonstrated focal choroidal excavation on spectral-domain OCT were collected. Their clinical characteristics and other features were also collected and analyzed.

**Results:**

In total, 42 focal choroidal excavations were identified in 31 patients, including 25 unilateral and 6 bilateral (37 eyes). The abnormal changes in these eyes with choroidal excavation were more prominent at the outer part of the neuro-retina, the retinal pigment epithelium (RPE) and the choroid. The average transverse diameter and depth of the excavations were 670.8 μm and 106.9 μm, respectively. In addition to the conforming and nonconforming types, the excavations could also be classified into 2 types according to their shape: type 1 – small with a sharp, cut-down contour; and type 2 – slightly larger with a gradual edge. The transverse diameter/depth ratio of the two types were significantly different (type1: 4.57 ± 1.65, type 2: 10.0 ± 5.2; p = 0.000). Four central serous chorioretinopathy (CSCR) cases were confirmed by fluorescein angiography; in these cases, the retinal detachment was larger than the area of excavation, and the inner segment/outer segment (IS/OS) and external limiting membrane (ELM) were above those of the normal part. Concomitant CNV was also found in another 2 cases.

**Conclusions:**

Focal choroidal excavation was not uncommon in Chinese patients. The choroid and the RPE at the excavation were impaired or vulnerable to other damage. Additionally, OCT might be useful in the differentiation between nonconforming excavations and ones with CSCR.

## Background

Focal choroidal excavation, an idiopathic condition that is localized at the macula, has been recently described [1]. Excavations have been categorized into two types: the conforming type, in which the outer retinal layers conform to retinal pigment epithelial alterations within the excavation; and the nonconforming type, in which a separation between the outer retina and the retinal pigment epithelium within the excavation exists [2]. Though it has been hypothesized that the nonconforming type might be caused by traction [2], the true cause of the nonconforming type of excavation is still not fully understood. However, this abnormality is frequently associated with central serous chorioretinopathy (CSCR), in which a localized retinal detachment is also present. Thus, the differentiation between the nonconforming type of excavation and the types associated with CSCR is important. In this study, a large series of focal choroidal excavations was reported, and the characteristics of these abnormalities were evaluated. Special attention was given to the characteristics of excavations with CSCR.

## Methods

This was a retrospective, consecutive, observational case study. The study adhered to the tenets of the Declaration of Helsinki. The study was approved by the Ethics Committee of the Eye & ENT Hospital of Fudan University.

### Data collection

Thirty-one patients with focal choroidal excavation were identified. Their medical records were reviewed, and the following information was collected: personal medical history, best-corrected visual acuity (BCVA), and the results of an ophthalmic clinical examination. The BCVA were measured using a standard Snellen chart and converted into LogMAR for further analysis. Fundus biomicroscopy was performed using a slit-lamp with a noncontact fundus lens (SuperField lens, Volk Optical Inc, Mentor, OH, USA). OCT was performed with a Cirrus HD-OCT (Carl Zeiss Meditec, Dublin, CA, USA), and fluorescein angiography (FFA) was performed with a TRC-50 DX fundus camera (Topcon, Tokyo, Japan).

**Figure 1:**
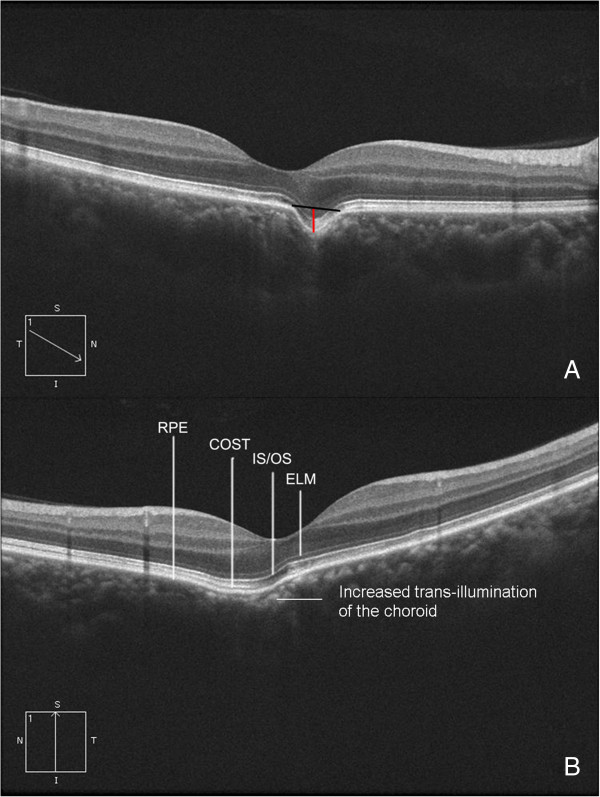
**Spectral-domain optical coherence tomography (SD-OCT) and dimension measurement of a focal choroidal excavation. (A)** A small depression slightly nasal to the fovea was present on the OCT image (type 1). The red and black lines show how the depth (red) and the diameter (black) were measured. **(B)** A shallow and gradual-edged excavation was present at the temporal of the fovea (type 2). The external limiting membrane (ELM), inner segment/outer segment (IS/OS), and cone outer segment tips (COST) follow the contour of the choroidal excavation, but the COST line discontinued at the fovea. The reflection of the choroid at the excavation is stronger than that from around.

All of the OCT images were obtained through a dilated pupil using 5 Line Raster scanning protocols with a 250-μm space between each line. During further analysis, special attention was given to the characteristics of the focal choroidal excavations. The height and width of each excavation was measured using internal caliper software with images crossing the center of the excavation (Figure 1). The excavations were categorized into two types according to their contour: type 1: small with a sharp cut-down contour, type 2: slightly larger with a gradual edge (Figure 1). The decision was made by two doctors (Dr Guo J and Dr Zhou X) separately, if there was disagreement then an experienced doctor (Dr Jiang CH) made the final decision.

The data were presented as means ± SD. The dimensions of different types of excavations were compared using a nonparametric test.

## Results

From June 2008 to December 2012, 31 patients (37 eyes, 15 males and 16 females) with focal choroidal excavation visited the Eye & ENT Hospital of Fudan University, Shanghai (Additional file 1: Table S1). The mean patient age was 39.6 ± 11.3 years (range 21 to 69 years), and the mean visual acuity (VA) was 0.17 ± 0.2 (logMAR). The disorder was unilateral in 25 patients and bilateral in 6 patients. In total, 15 patients (18 eyes) were asymptomatic and were found to have choroidal excavations during routine general exams; 14 patients (17 eyes) complained of gradually blurred vision. Another 2 patients (2 eyes) received an OCT exam because of CSCR in the other eye. Except for a traumatic cataract (a history of trauma 10 years ago) in 1 eye and CSCR in 2 other eyes, the ophthalmic histories and exams of all fellow eyes in the 22 unilateral cases were negative, with no special findings.

**Figure 2:**
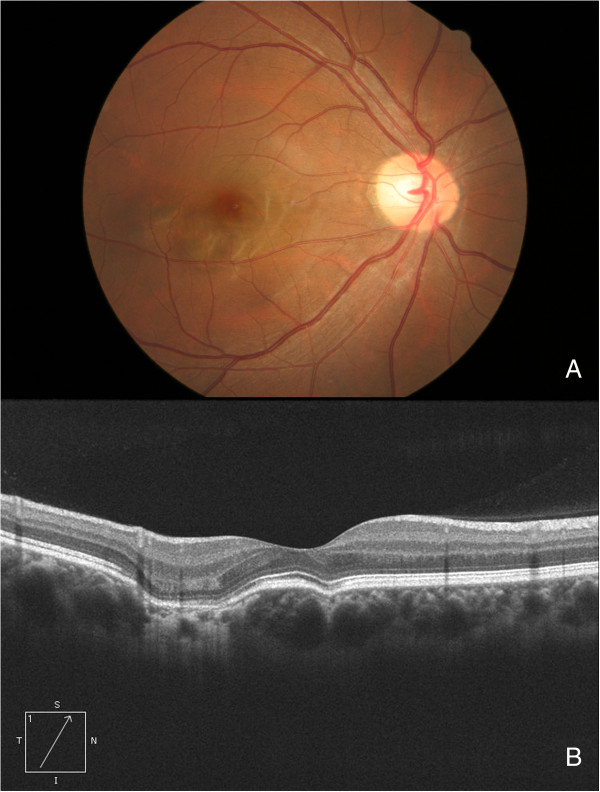
**Fundus image and OCT of an eye with multiple excavations.** These images are from a 29-year-old female who underwent a routine general exam. **(A)** The fundus appeared even and normal. **(B)** Two excavations were present on the OCT.

**Figure 3:**
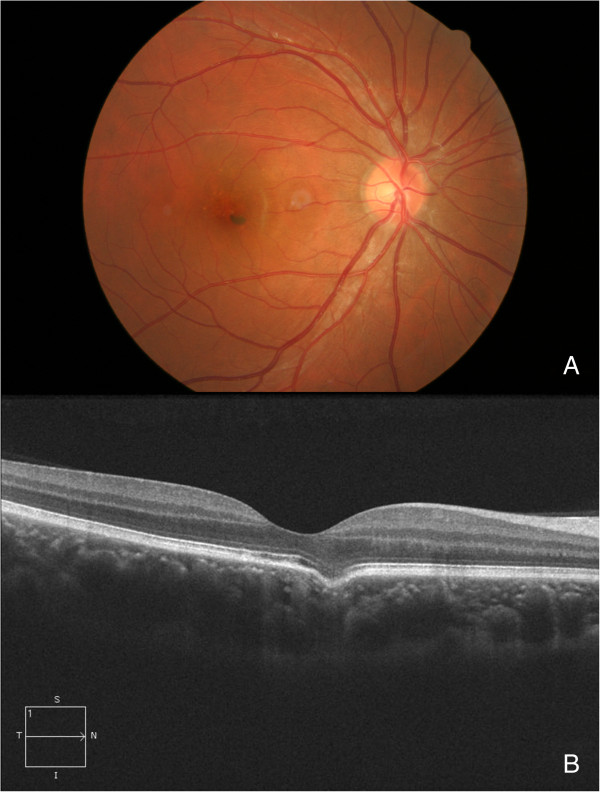
**Fundus image and OCT of an eye with a solitary excavation.** These images are from a 30-year-old male who underwent a routine general exam. **(A)** There were some pigmentary changes at the macula. **(B)** An excavation at the fovea was present on the OCT.

Of the 37 eyes, 15 were emmetropic, and 22 were myopic −4.70 ± 2.9D (range −0.75D to -12D). In the majority of patients without CSCR or choroidal neovascularization (CNV), the refraction in both eyes was similar (difference within 1.5D). However, in 1 patient with normal vision, a unilateral lesion was located in the center of the fovea; this eye was more myopic than the fellow eye (with focal choroidal excavation −5.75D, fellow -4D). A similar finding was noted in another patient with bilateral focal choroidal excavation (emmetropic in the right eye with parafoveal excavation, −3.5D in the left eye with subfoveal excavation). The fundus of the 37 eyes could be quite normal (Figure 2) or have few pigmentary changes (Figure 3).

All of the patients received OCT exams bilaterally, with the exception of 1 patient who had a traumatic cataract in the fellow eye. On OCT, the choroidal excavation was typically a local depression of the choroid at or near the fovea. The excavation was solitary in 33 eyes and multiple in 4 eyes (Figures 1 and 2); in total, 42 (1 eye had 3 focal choroidal excavations) excavations were identified. All were located in the macular area.

Excavations in 27 (73% of all) eyes were of the conforming type, 8 (21.6%) with local retinal detachment (likely 7 CSCR and 1 nonconforming), and 2 (5.4%) had CNV. Among the 42 excavations, 24 (57%) were type 1, and 18 (43%) were type 2. The average transverse diameter of the 42 excavations was 670.8 ± 386.3 μm (range 97 to 1758 μm), and the average depth was 106.9 ± 46.8 μm (range 24 to 218 μm). The transverse diameter of the type 1 was smaller than that of the type 2 (type 1: 576.1 ± 351.2 μm, type 2: 802.3 ± 402.9 μm, p = .02, nonparametric test); however, the depth was similar between the two types (type 1: 118.1 ± 49.9 μm, type 2: 91.3 ± 38.2 μm μm, p = .1). The ratio (transverse diameter/depth) was calculated, the ratio of type 1 was 4.57 ± 1.65 and type 2 10.0 ± 5.2, significantly different was found between the two types (p = 0.000). The outer border of the choroid at the choroidal excavation area did not change to a large extent (Figures 1, 2 and 3), but the inner border was located further outward than the other part of the macula; additionally, on OCT there was increased trans-illumination of the choroid at the excavation area (Figure 1).

**Figure 4:**
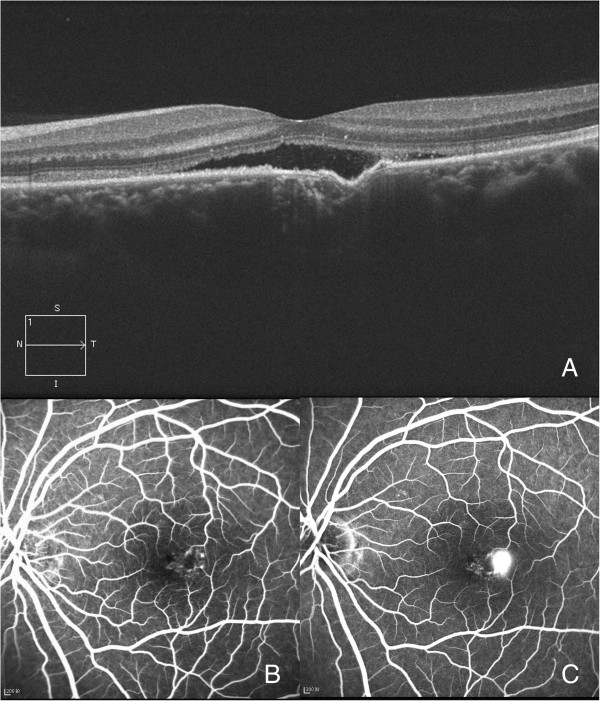
**Focal choroidal excavation with concomitant central serous chorioretinopathy (CSCR).** These images are from a 32-year-old male who was diagnosed with CSCR. **(A)** An excavation was present with localized retinal detachment. **(B** and **C)** An early window defect with later leakage within the excavation was found by FFA (B-early stage, C-later stage).

**Figure 5:**
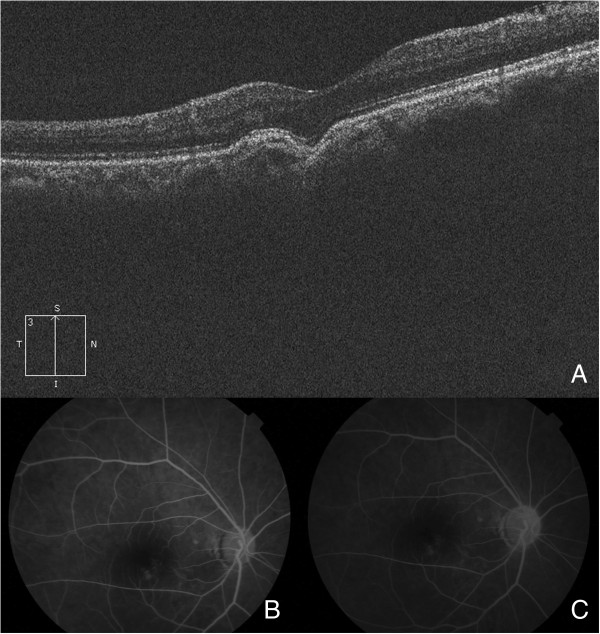
**Excavation with retinal pigment epithelium (RPE) changes.** These images were from a 56-year-old female who complained of decreased vision and was diagnosed with a cataract. **(A)** On OCT, the RPE at the lower edge of the excavation (left part) was slightly elevated. **(B** and **C)** On FFA, a window defect was present; it corresponded with the PRE change on OCT (B-early stage, C-later stage).

In the 27 eyes with conforming-type excavations, the inner part of the retina over the choroidal excavation appeared rather normal. However, slight changes were identified in the outer retina. The outer nuclear layer (ONL) over the choroidal excavation appeared to be slightly thicker than that of the other part. Though the finer bands of the external limiting membrane (ELM) and the inner segment/outer segment (IS/OS) followed the contour of the choroidal excavation, there was always some defect in the line representing the cone outer segment tips (COST) (Figure 1). In the 8 eyes with local retinal detachment, the thickening of the ONL was not as obvious (Figure 4). A leaking spot within the excavation was observed in 4 patients who underwent FFA (Figure 4). Of these, 3 patients had type 2 excavations and one had type 1; their vision was 0.3-0.52 (logMAR). In these 4 eyes, the localized retinal detachment was larger than the area of excavation, and the fine lines of the ELM and the IS/OS at the detached area were slightly above the same layer from the normal part (Figure 4). At some area (either within or at the edge of the choroidal excavations), the line representing the RPE looked irregular on OCT. However, this type of RPE change was also observed in an eye of conforming type without CSCR and corresponded with a window defect on FFA (Figure 5).

**Figure 6:**
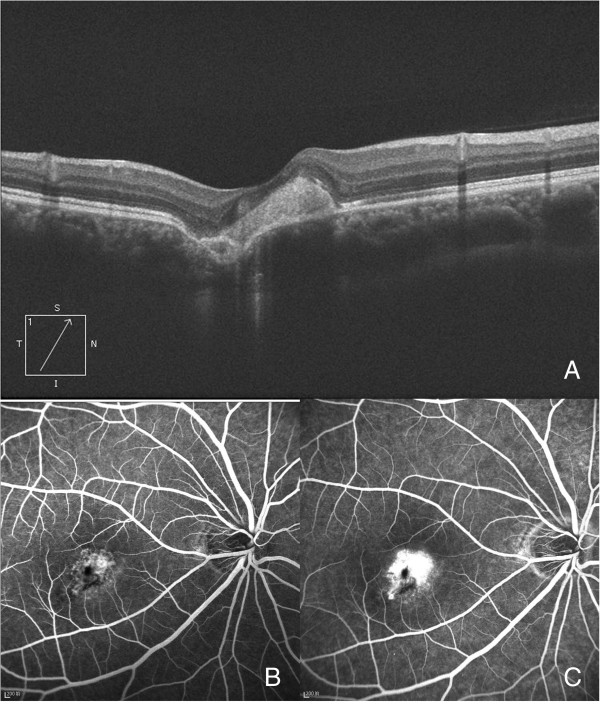
**Excavation with concomitant choroidal neovascularization (CNV).** These images are from a 30-year-old female who was diagnosed with CNV. **(A)** A focal area of hyper-reflectivity above the RPE band involving the excavation and a localized RPE detachment within the excavation were present on OCT. **(B** and **C)** These findings corresponded with the early vascular network and late leakage on FFA (B-early stage, C-later stage).

In the 2 cases with concomitant CNV, a mass located under the neural-retina and partly within the excavation was identified. In addition, FFA revealed an area with high fluorescein signal at an early stage and gradual fluorescein leakage at a later stage (Figure 6).

## Discussion

In this report, a large series of focal choroidal excavation cases in Chinese patients were collected, and their clinical and OCT findings were analyzed. Our series included 31 Chinese patients. Former reports noted that Asians were included in their series [2-7]; thus, this condition might be more common in Asia, or the incidence may be due to our large population. The average patient age was 39.6 ± 11.3 years, and the patients’ medical histories indicated normal vision. Nearly half of the patients (15/31, 48%) were asymptomatic and had a BCVA better than 16/20(logMAR 0.1). The visual symptoms in the other patients had developed quite recently, which suggested that their eyes had been normal for a long period of time. Also Ellabban A et al. did not observe any change in the focal choroidal excavation during the follow-up [8]. These findings supported the hypothesis proposed by Ron Margolis et al. that this abnormality might be congenital [2]. On the other hand, recently Savastano MC et al. found a positive correlation between Epstein-Barr virus infection and focal choroidal excavation, and Lee CS reported the changes of focal choroidal excavation in their observation [9,10], so further study is required to reveal the etiology of this entity.

Thickening of the ONL was identified at the choroidal excavation area. With a small defect at the choroid, the adhesion between the RPE and the photoreceptors that normally keep them attached could pull the retina outward, whereas the inner retina or the vascular system may attempt to hold the retina in place. Therefore, these 2 forces might result in ONL thickening. And this force is greatly reduced, if not completely absent, in nonconforming-type eyes or in eyes with CSCR, in which the retina is detached at the excavation area. This also explained why the thickening of the ONL was not prominent in these eyes.

The OCT images and the width and depth of the focal choroidal excavation in our series were similar to those of others [10,11]. In addition to the conforming and nonconforming types of excavations, we found that focal choroidal excavations could also be divided into 2 types by their contours. And images from other reports also supported this (type 1: Figure 1; type 2: Figure 5 all in reference [2]). The force of pulling the retina inwards in the posterior-anterior direction might be more prominent or focused in eyes with type 1 excavations, in which the ratio (transverse diameter/depth) is much smaller, suggesting the punch-out to be more sudden. This force might contribute to the separation between the outer retina and the RPE within the excavation in the nonconforming type. The nonconforming cases that Ron Margolis, John C. Chen, Suzuki M and Ellabban A demonstrated were type 1 supported the suggestion that the force pulling the retina inwards was the cause of the nonconforming type [2,3,8,12].

The incidence of CSCR in our series is much higher than the 10/1,000,000 reported in the literature [13]. Others have reported focal choroidal excavation with CSCR, but the characteristics of these CSCR cases and the differentiation from the nonconforming types has not been well described. Because both nonconforming-type and CSCR excavations present with local retinal detachment, this might make diagnosis difficult. Although FFA helps with differentiation, not all CSCR eyes have a positive leaking point [14]. As a result, other methods should be evaluated, and OCT might be of value. In our series, FFA confirmed acute CSCR in 4 eyes. By comparing these 4 eyes with the nonconforming-type cases in the literature [2,3,5,15], the following differences were noted: first, the retinal detachment was always within the excavation in nonconforming cases, while it usually exceeded the excavation in CSCR cases; second, the fine lines of the ELM and the IS/OS at the detached area were slightly above the normal counterparts in CSCR cases, while they were below those of the normal counterparts in nonconforming cases; third, changes in PRE were more common and prominent in CSCR cases; and fourth, CSCR eyes could be either type 1 or 2 (there were more type 2 in our series), while nonconforming-type cases described in previous reports were mostly type 1 [2,3,5,15]. And since recent study found that choroidal thickness was increased in focal choroidal excavation patients if complicated by CSC, so this could be another point that could be helpful in the differentiating [12]. Additionally, the BCVA was usually worse in CSCR cases. It was hypothesized that traction might contribute to the nonconforming type and that this force could never exceed the boundary of the excavation; thus, the retinal detachment in the nonconforming type was limited to the excavation, and the ELM or the IS/OS line was below that from the normal eye. However, in CSCR, the fluid is due to leakage; as a result, the detachment could be larger than the excavation, and the fine lines of the ELM and the IS/OS could be more elevated.

CNV was also identified in 2 other patients. The pathogenesis of both CSCR and CNV ranges from a basic alteration in the choroid to an involvement of the RPE [13,16-18]; the high incidence in our and former reports [2,6,15] paired with the fact that the leaking points in our CSCR and CNV eyes were all within the excavations suggested that the choroid and the RPE at the choroidal excavation were impaired or at least vulnerable to other damage. In the former reports, changes of RPE and abnormal choroidal circulation at focal choroidal excavation were found [8,12]. So abnormality at the level of RPE or choroid might contribute to the pathogenesis of CSC or CNV in these patients.

There are more type 2 excavations in our CSCR cases. Type 2 excavations are larger than type 1 excavations; large excavations might imply that more choroidal tissue and RPE are involved and thus more easily develop such complications. However, even in cases without CSCR, the trans-illumination of the choroid was increased, and abnormality of the RPE was identified. All of these findings suggest that the RPE at that specific area might be impaired. Thus, patients with focal choroidal excavation should be followed even if they are asymptomatic. It is interesting to note that we have 2 conforming cases with CSCR in the otherwise healthy fellow eyes. Future study with more cases and a longer follow-up period might be necessary.

## Conclusion

The OCT and other characteristics of focal choroidal excavations identified in a group of Chinese patients were analyzed. However, this is only a cross-sectional study. Further study is required to improve our knowledge about the pathogenesis and natural history of this condition.

## Abbreviations

OCT: Optical coherence tomography; RPE: Retinal pigment epithelium; CSCR: Central serous chorioretinopathy; IS/OS: The inner segment/outer segment; ELM: External limiting membrane; BCVA: Best-corrected visual acuity; FFA: Fluorescein angiography; CNV: Choroidal neovascularization; ONL: Outer nuclear layer; COST: Cone outer segment tips.

## Competing interests

The authors have no financial competing interests.

## Authors’ contributions

All authors conceived of and participated in the study design. All authors participated in the eye examinations and data collection. JG, CJ, LZ and XZ performed the statistical analysis and drafted the manuscript. CJ, GX, WW and YW did critical revision of the manuscript for important intellectual content. All authors read and approved the final manuscript.

## Pre-publication history

The pre-publication history for this paper can be accessed here:

http://www.biomedcentral.com/1471-2415/14/63/prepub

## Supplementary Material

Additional file 1: Table S1 The demography of patients with focal choroidal excavation. Click here for file
