# Supplementary material for: Clinical and optic coherence tomography findings of focal choroidal excavation in Chinese patients
Source: BMC Ophthalmol. 2014 May 6;14:63. doi: 10.1186/1471-2415-14-63 (PMC4107723; doi:10.1186/1471-2415-14-63)
Supplement: Additional file 1: Table S1 — The demography of patients with focal choroidal excavation. [file 1471-2415-14-63-S1.doc]

**Additional file 1 Table S1 The demography of patients with focal choroidal excavation**

| **No** | **Gender** | **Age (y)** | **Eye** | **BCVA^a^ (logMAR)** | **Refraction(D)** | **Clinical state** | **FCE types** | **number of FCE** | **FCE types based on shape** |
| --- | --- | --- | --- | --- | --- | --- | --- | --- | --- |
| 1 | F | 50 | OD | 0.00 | -5.75 | asymptomatic | conforming | 2 | 2 type1 |
| 2 | M | 21 | OS | 0.00 | -4 | asymptomatic | conforming | solitary | type1 |
| 3 | F | 41 | OD | 0.10 | -10 | asymptomatic | conforming | solitary | type1 |
| 4 | F | 46 | OS | 0.00 | 0 | asymptomatic | conforming | solitary | type2 |
| 5 | F | 36 | OD | 0.00 | 0 | asymptomatic | conforming | solitary | type2 |
| 6 | F | 36 | OD | 0.00 | 0 | asymptomatic | conforming | solitary | type1 |
| 7 | M | 37 | OD | 0.00 | -5 | asymptomatic | conforming | solitary | type2 |
|  |  |  | OS | 0.00 | -5 | asymptomatic | conforming | 3 | 1 type1,2 type2 |
| 8 | F | 40 | OS | 0.00 | 0 | asymptomatic | conforming | solitary | type2 |
| 9 | M | 30 | OD | 0.05 | -3 | asymptomatic | conforming | solitary | type1 |
| 10 | F | 29 | OD | 0.00 | 0 | asymptomatic | conforming | 2 | 1 type1，1 type2 |
|  |  |  | OS | 0.15 | -3.5 | asymptomatic | conforming | 2 | 1 type1，1 type2 |
| 11 | F | 35 | OD | 0.00 | -2 | asymptomatic | conforming | solitary | type1 |
| 12 | F | 32 | OS | 0.05 | -3 | asymptomatic | conforming | solitary | type1 |
| 13 | M | 40 | OD | 0.00 | -3.25 | asymptomatic | conforming | solitary | type1 |
|  |  |  | OS | 0.00 | -3.25 | asymptomatic | conforming | solitary | type1 |
| 14 | F | 27 | OS | 0.00 | -1 | asymptomatic | conforming | solitary | type1 |
| 15 | M | 32 | OD | 0.10 | -5 | asymptomatic | conforming | solitary | type2 |
| 16 | F | 44 | OS | 0.40 | -0.75 | blurred vision | conforming | solitary | type2 |
| 17 | F | 69 | OD | 0.22 | -7.5 | blurred vision | conforming | solitary | type1 |
| 18 | M | 63 | OD | 0.22 | -10.5 | blurred vision | conforming | solitary | type1 |
|  |  |  | OS | 0.22 | -12 | blurred vision | conforming | solitary | type1 |
| 19 | F | 56 | OD | 0.52 | 0 | blurred vision | conforming | solitary | type1 |
| 20 | F | 59 | OS | 0.00 | 0 | fellow eye blurred vision | conforming | solitary | type2 |
| 21 | M | 39 | OD | 0.10 | 0 | fellow eye blurred vision | conforming | solitary | type1 |
| 22 | M | 52 | OD | 0.00 | -4 | blurred vision | conforming | solitary | type2 |
|  |  |  | OS | 0.00 | -3 | blurred vision | Nonconforming suspected | solitary | type1 |
| 23 | M | 28 | OS | 0.15 | -4 | blurred vision | CSCR(suspected) | solitary | type2 |
| 24 | M | 43 | OD | 0.52 | 0 | blurred vision | CSCR(suspected) | solitary | type2 |
| 25 | M | 41 | OS | 0.52 | 0 | blurred vision | CSCR（confirmed by FFA） | solitary | type2 |
| 26 | F | 27 | OD | 0.40 | 0 | blurred vision | CSCR（confirmed by FFA） | solitary | type2 |
| 27 | M | 30 | OS | 0.30 | 0 | blurred vision | CSCR（confirmed by FFA） | solitary | type2 |
| 28 | M | 32 | OS | 0.30 | 0 | blurred vision | CSCR（confirmed by FFA） | solitary | type1 |
| 29 | M | 40 | OD | 0.40 | 0 | blurred vision | CSCR(suspected) | solitary | type1 |
| 30 | F | 30 | OD | 0.40 | 0 | blurred vision | CNV（confirmed by FFA） | solitary | type1 |
| 31 | M | 43 | OD | 0.70 | -4 | blurred vision | CNV（confirmed by FFA） | solitary | type1 |
|  |  |  | OS | 0.00 | -4 | blurred vision | conforming | solitary | type2 |
